# Capsanthin inhibits migration and reduces N-linked glycosylation of PD-L1 via the EZH2-PD-L1 axis in triple-negative breast cancer brain metastasis

**DOI:** 10.1038/s41420-025-02368-1

**Published:** 2025-03-04

**Authors:** Yi-Chung Chien, Jia-Yan Wu, Liang-Chih Liu, Yung-Luen Yu

**Affiliations:** 1 Institute of Translational Medicine and New Drug Development, China Medical University, Taichung, Taiwan; 2 Center for Molecular Medicine, China Medical University Hospital, Taichung, Taiwan; 3 School of Medicine, College of Medicine, China Medical University, Taichung, Taiwan; 4 Department of Surgery, China Medical University Hospital, Taichung, Taiwan; 5 Department of Medical Laboratory Science and Biotechnology, Asia University, Taichung, Taiwan

**Keywords:** Breast cancer, Drug development

## Abstract

Breast cancer metastasis to the brain, occurring in about 15–25% of cases, represents a major obstacle in the treatment of triple-negative breast cancer (TNBC). The molecular mechanisms driving this form of metastasis are still largely unknown. PD-L1, an immune checkpoint protein, is central to tumor immune evasion and has become a focus for immunotherapy development. While PD-L1 inhibitors have shown success in various cancer types, their effectiveness in TNBC brain metastases remains to be fully investigated. This highlights the urgent need to understand the complex interactions between metastatic brain tumors and the tumor microenvironment in TNBC patients. Gaining insights into these dynamics is crucial for developing new targeted therapies, including those that modulate the PD-L1 pathway, to better manage and treat TNBC brain metastases. We explore the impact of Capsanthin on the tumor microenvironment of brain metastases in triple-negative breast cancer (TNBC). Our results reveal that Capsanthin effectively inhibits the migration of brain metastasis TNBC cells. Furthermore, Capsanthin significantly reduces the expression of EZH2 and N-linked glycosylated PD-L1 proteins and mRNA in TNBC cells, encompassing both primary and metastatic sites, as well as in mesenchymal stem cells (3A6). Data from The Cancer Genome Atlas (TCGA) indicate that elevated expression levels of EZH2 correlate with poorer patient prognosis. Immunoprecipitation assays demonstrate a direct interaction between EZH2 and PD-L1 in brain metastases of TNBC, underscoring the pivotal role of the EZH2-PD-L1 axis. Additionally, Capsanthin was found to suppress the expression of epithelial-mesenchymal transition (EMT) markers in metastatic brain TNBC cells and mesenchymal stem cells. Our results suggest that Capsanthin can modulate the tumor microenvironment and inhibit key pathways involved in cancer progression, offering potential therapeutic benefits for patients with TNBC brain metastases.

## Introduction

Breast cancer brain metastasis (BCBM) is a critical and challenging complication, affecting approximately 15–25% of breast cancer patients [1]. TNBC, characterized by the lack of estrogen receptors, progesterone receptors, and HER2 expression, is particularly prone to brain metastasis. This subtype is known for its aggressive clinical course and poor prognosis, especially when brain metastases are involved [2, 3]. Patients with TNBC brain metastases have a significantly reduced median survival, often ranging from 3 to 7 months after diagnosis. Current treatment strategies for BCBM include surgery, radiation therapy, and systemic chemotherapy. Surgical resection is typically reserved for patients with a limited number of accessible brain lesions, but its invasiveness and potential for morbidity limit its use. Radiation therapy, including whole-brain radiation therapy (WBRT) and stereotactic radiosurgery (SRS), provides symptomatic relief and local control but is often associated with neurocognitive decline and does not significantly improve overall survival [4–7]. Therefore, there is an urgent need for innovative strategies that can effectively target TNBC brain metastases and improve patient outcomes.

The tumor microenvironment (TME) of brain metastases from breast cancer is characterized by a complex interplay of immune cells, stromal components, and tumor cells. This microenvironment plays a crucial role in tumor progression, immune evasion, and resistance to therapy. Recent studies have highlighted the highly immunosuppressive nature of the TME in BCBM, posing significant challenges for effective treatment [8]. One of the key components of the immunosuppressive TME is the immune checkpoint protein PD-L1 (programmed death-ligand 1). PD-L1 is expressed on the surface of tumor cells and interacts with the PD-1 receptor on T cells, leading to the inhibition of T cell-mediated immune responses. This interaction allows tumor cells to evade immune surveillance and proliferate unchecked, making PD-L1 a prime target for immunotherapy [9, 10]. N-linked glycosylation is a common post-translational modification of PD-L1. The heavily glycosylated form of PD-L1, featuring N-linked glycans, is observed in various cancer types and exhibits distinct patterns in Western blot analyses. In contrast, the non-glycosylated form of PD-L1 typically appears at approximately 33 kDa [11]. Immune checkpoint inhibitors targeting the PD-1/PD-L1 axis, such as pembrolizumab and nivolumab, have shown promise in reactivating the immune system to recognize and destroy tumor cells in various cancers. However, the effectiveness of PD-L1 inhibitors in treating TNBC brain metastases remains an area of active investigation.

Capsanthin, a natural carotenoid found in red peppers, has gained significant attention for its potent antioxidant and anti-inflammatory properties. Beyond its role as a dietary antioxidant, emerging evidence suggests that Capsanthin possesses notable anti-cancer effects, inhibiting the proliferation and migration of various cancer cell types [12–15]. Capsanthin exerts its anti-cancer effects through multiple mechanisms. It modulates oxidative stress pathways, reducing reactive oxygen species (ROS) levels within tumor cells, which in turn inhibits tumor cell proliferation and induces apoptosis [15]. In the context of TNBC and its brain metastases, Capsanthin has shown potential in modulating the tumor microenvironment. Studies have indicated that Capsanthin can reduce the expression of critical proteins involved in tumor development, such as EZH2. EZH2, a histone methyltransferase, is often overexpressed in TNBC and is associated with poor prognosis. By downregulating EZH2 expression, Capsanthin can inhibit tumor growth [13]. Previous studies have demonstrated that EZH2, an epigenetic regulator, is significantly upregulated in aggressive cancer subtypes, including TNBC, and is associated with poor prognosis and metastatic potential. For instance, recent research has shown that EZH2-mediated H3K27 trimethylation contributes to immune evasion by downregulating T cell activating molecules, thus promoting metastasis to immune-privileged sites such as the brain [16, 17]. Furthermore, EZH2 has been identified as a critical driver in reprogramming the tumor microenvironment, making it an appealing therapeutic target. Therefore, Capsanthin represents a multifaceted therapeutic agent with the potential to address critical challenges in the treatment of TNBC and its brain metastases. Its ability to modulate oxidative stress, inhibit key signaling pathways, and alter the immune microenvironment underscores its promise as a novel therapeutic approach. Further research and clinical trials are warranted to fully elucidate the potential of Capsanthin in cancer therapy and to optimize its integration into current treatment paradigms.

## Results

### *EZH2* level correlates with survival of grade 3 TNBC patients

**Fig. 1:**
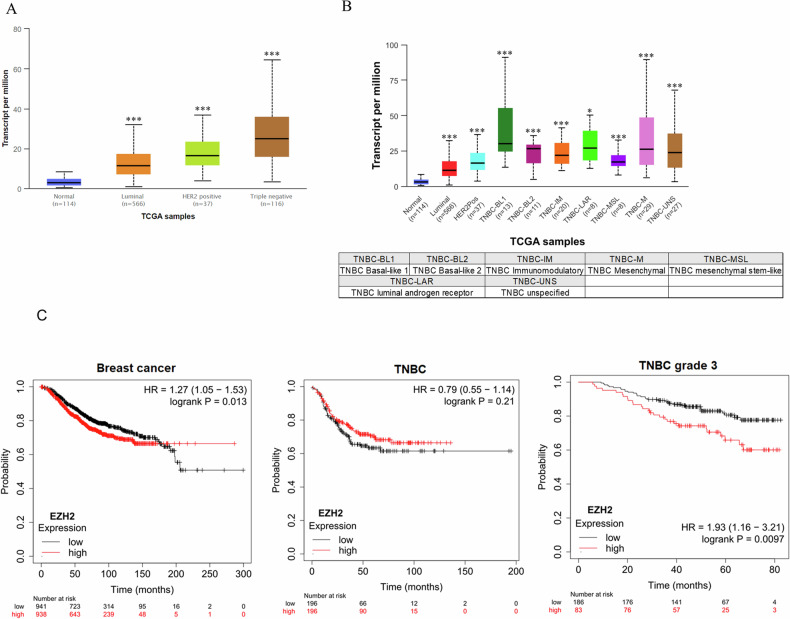
The expression of EZH2 in different types of breast cancer patients. **A** Box plots showing the transcript per million (TPM) values of EZH2 in normal breast tissue (*n* = 114), luminal breast cancer (*n* = 566), HER2 positive breast cancer (*n* = 37), and triple-negative breast cancer (TNBC, *n* = 116) from TCGA samples. EZH2 expression is significantly higher in luminal, HER2 positive, and TNBC samples compared to normal breast tissue. **B** Box plots displaying EZH2 expression in different subtypes of TNBC, including basal-like 1 (BL1, *n* = 16), basal-like 2 (BL2, *n* = 10), immunomodulatory (IM, *n* = 9), mesenchymal (M, *n* = 5), mesenchymal stem-like (MSL, *n* = 5), luminal androgen receptor (LAR, *n* = 6), and unspecified (UNS, *n* = 27). EZH2 expression is significantly higher in these subtypes compared to normal breast tissue. **C** Kaplan–Meier survival curves illustrating the relationship between EZH2 expression and overall survival in breast cancer patients. Left: High EZH2 expression is associated with poorer survival in all breast cancer patients (HR = 1.27, 95% CI = 1.05–1.53, *p* = 0.013). Middle: There is no significant difference in survival between high and low EZH2 expression in TNBC patients (HR = 0.79, 95% CI = 0.55–1.14, *p* = 0.21). Right: High EZH2 expression correlates with significantly poorer survival in grade 3 TNBC patients (HR = 1.93, 95% CI = 1.16–3.21, *p* = 0.0097) (*p < 0.05, **p < 0.01, ***p < 0.001).

To assess the clinical significance of *EZH2*, we analyzed publicly available data from The Cancer Genome Atlas (TCGA) database. The result suggests that *EZH2* expression is significantly higher in breast cancer tissues compared to normal breast tissues, with the highest levels observed in more aggressive subtypes like TNBC (Fig. 1A). However, further analysis revealed that the expression levels of *EZH2* are significantly higher in different subtypes of TNBC patients compared to normal individuals. These findings suggest that *EZH2* may play a crucial role in the progression of TNBC and within the tumor microenvironment (Fig. 1B). Moreover, in our analysis of the correlation between *EZH2* expression and patient survival, we observed an intriguing phenomenon. High *EZH2* expression was associated with significantly reduced survival rates, but this effect was only seen in late-stage TNBC patients (Grade 3). This correlation was not observed in the broader breast cancer patient population or among TNBC patients as a whole (Fig. 1C). These findings from the TCGA database suggest that EZH2 may function as an oncogene, facilitating the metastasis of breast tumors to the brain.

### Capsanthin inhibits the migration of TNBC cells

**Fig. 2:**
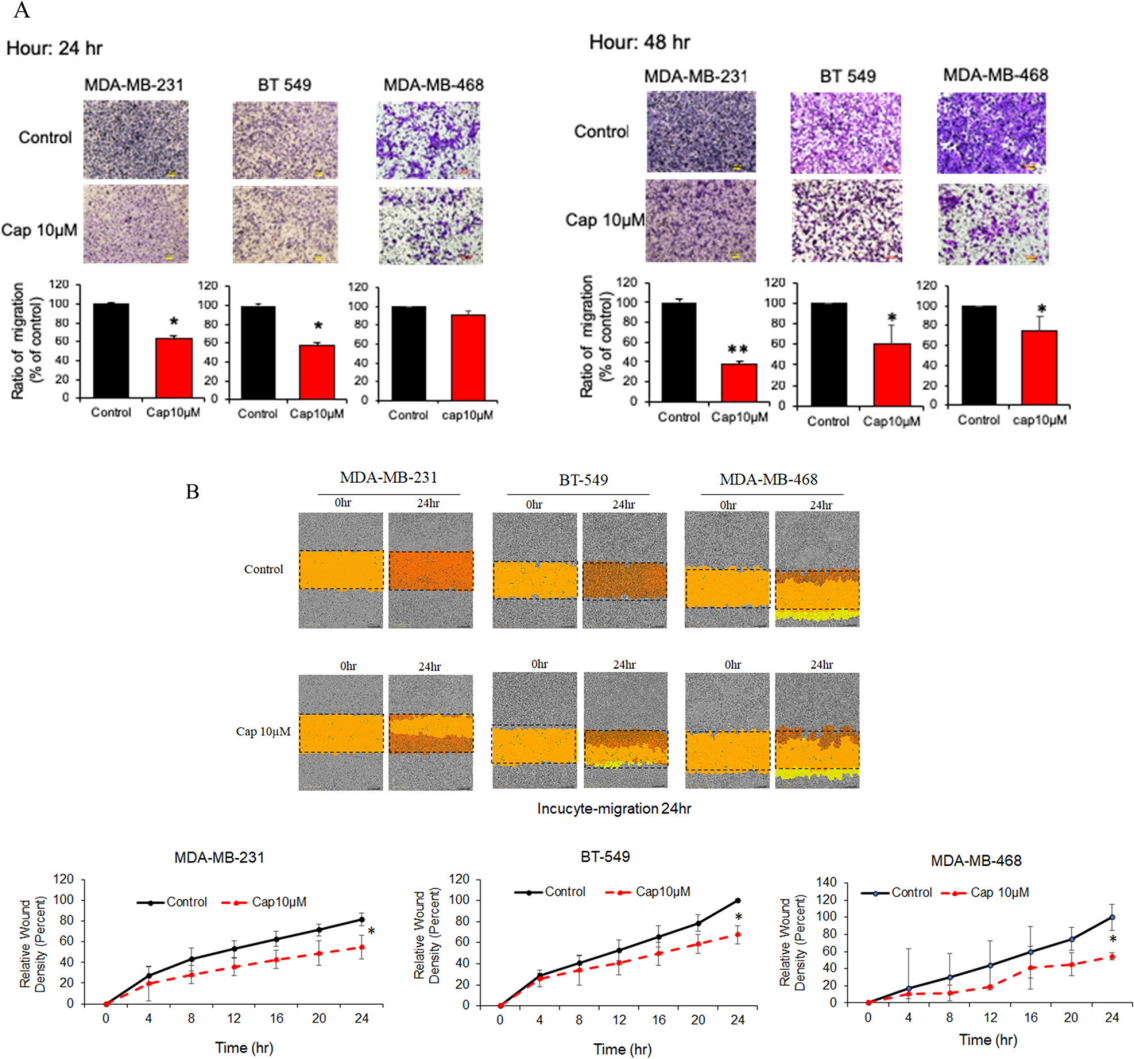
Capsanthin inhibits migration of TNBC cells. **A** Transwell migration assays show that Capsanthin treatment (10 μM) significantly reduces the migration of MDA-MB-231, BT549, and MDA-MB-468 TNBC cell lines. Migration was assessed at 24 h for MDA-MB-231 and BT549, and at 48 h for all three cell lines. Student’s t-test (*n* = 3). **B** Real-time imaging using the IncuCyte system confirms the inhibitory effect of Capsanthin on TNBC cell migration. Capsanthin treatment (10 μM) significantly reduced the migration of TNBC cells after 24 h. Student’s *t*-test (*n* = 3). (***p < 0.001).

Based on our previous research, we found that Capsanthin can inhibit the expression of EZH2 and suppress growth in TNBC [13]. Therefore, in this study, we further investigated whether Capsanthin can inhibit the metastatic potential of TNBC cells. Firstly, we utilized a transwell assay to evaluate the metastatic potential of three TNBC cell lines (MDA-MB-231, BT549, and MDA-MB-468) following treatment with 10 μM Capsanthin. As shown in the results, after 24 h of Capsanthin treatment, the metastatic ability of the two more invasive TNBC cell lines (MDA-MB-231 and BT549) was significantly inhibited. After 48 h of treatment, Capsanthin effectively suppressed the metastatic potential of all three cell lines (Fig. 2A). Next, we used the real-time imaging system, IncuCyte assay, to further confirm the ability of Capsanthin to inhibit TNBC cell migration. We observed that after 24 h of 10 μM Capsanthin treatment, the migration capacity of TNBC cells was effectively suppressed (Fig. 2B). Overall, these findings demonstrate that Capsanthin not only reduces EZH2 expression and growth in TNBC cells but also significantly inhibits their metastatic and migratory capabilities. This suggests that Capsanthin could be a promising therapeutic agent for managing TNBC, particularly in preventing its metastasis.

### Capsanthin inhibits migration and reduces EZH2 and EMT marker expression in highly invasive 231Br cells compared to primary MDA-MB-231 cells

**Fig. 3:**
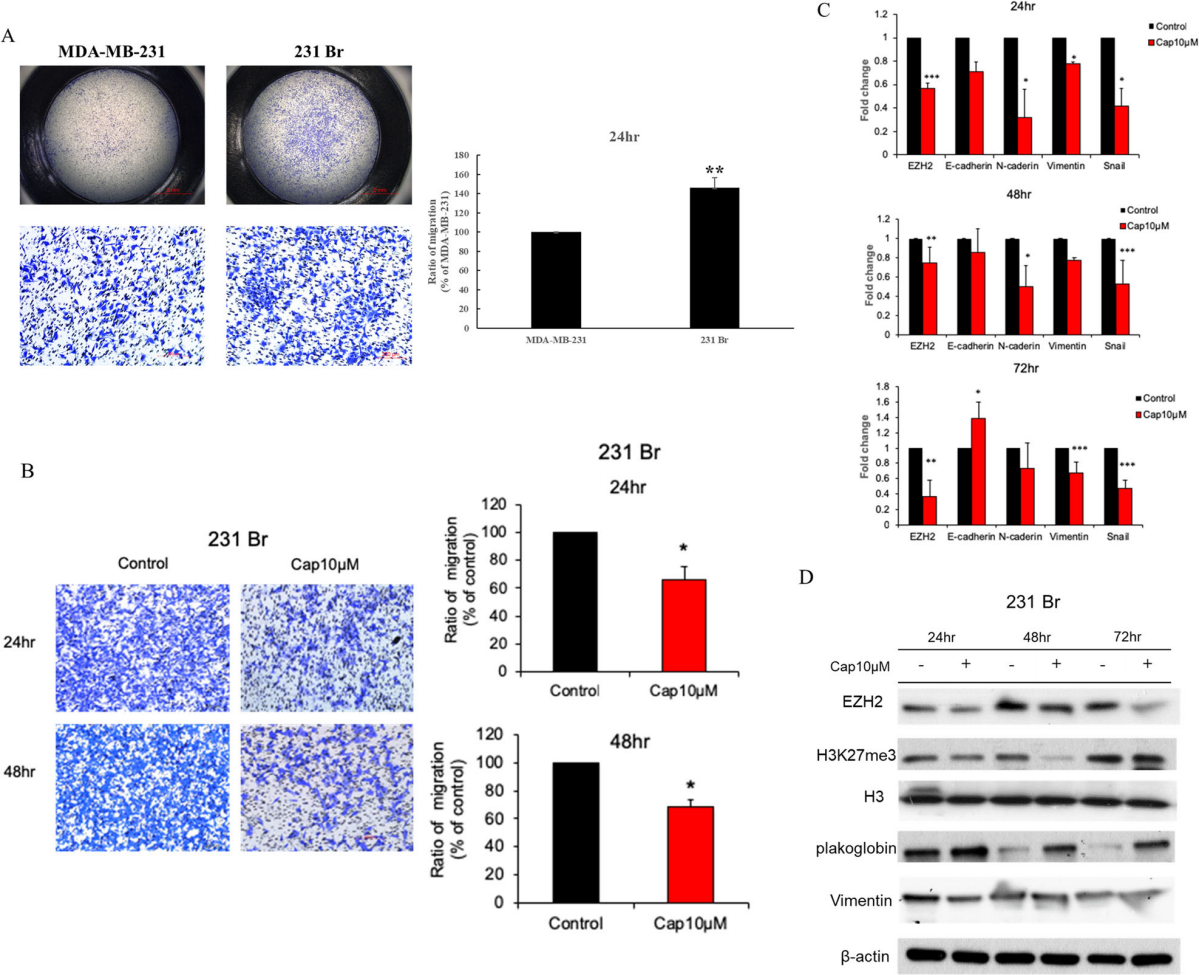
Capsanthin suppresses migration and EMT in 231Br Cells. **A** Transwell migration assays indicate that the brain metastatic TNBC cell line 231Br has significantly higher migration capabilities compared to the primary MDA-MB-231 cell line. Student’s *t*-test (*n* = 3). **B** Capsanthin treatment (10 μM) significantly inhibits the migration of 231Br cells after both 24 and 48 h. Student’s t-test (*n* = 3). **C** qRT-PCR analysis shows that Capsanthin treatment reduces the mRNA expression of EZH2 and EMT markers (E-cadherin, N-cadherin, Vimentin, Snail) in 231Br cells at 24, 48, and 72 h. Student’s *t*-test (*n* = 3). **D** Western blot analysis indicates that Capsanthin treatment reduces the protein levels of EZH2 and EMT markers (E-cadherin, N-cadherin, Vimentin, Snail) in 231Br cells at 24, 48, and 72 h. (*p < 0.05, **p < 0.01, ***p < 0.001).

To further investigate the potential molecular mechanisms underlying breast cancer brain metastasis, we established a brain-tropic metastatic cell line from MDA-MB-231, known as 231Br. This cell line possesses the capability to metastasize to the brain, enabling the study of specific factors that contribute to this process. First, we illustrates that the brain metastatic TNBC cell line, 231Br, exhibits significantly higher migration capabilities compared to the primary TNBC cell line, MDA-MB-231. This is evident from the increased number of cells migrating through the transwell membrane after 24 h, as quantified in the accompanying bar graph (Fig. 3A). Our result also demonstrates that treatment with 10 μM Capsanthin for 24 and 48 h significantly inhibits the migration of 231Br cells. The transwell migration assay results show a notable decrease in cell migration following Capsanthin treatment compared to the control, with quantification confirming a significant reduction in the migration ratio at both time points (Fig. 3B). Next, we further present the effects of Capsanthin on the expression of EZH2 and EMT markers in 231Br cells. Capsanthin treatment led to a significant downregulation of EZH2, E-cadherin, N-cadherin, Vimentin, and Snail mRNA levels at 24, 48, and 72 h. These changes suggest that Capsanthin effectively inhibits key regulators of EMT in these cells (Fig. 3C). Notably, the result shows the Western blot analysis of protein expression levels in 231Br cells treated with Capsanthin for 24, 48, and 72 h. Capsanthin treatment resulted in reduced levels of EZH2, H3K27me3, plakoglobin, and Vimentin, further supporting the inhibition of EMT markers at the protein level (Fig. 3D). These results indicate that Capsanthin not only reduces the expression of EZH2 but also suppresses the EMT process, thereby potentially decreasing the metastatic potential of 231Br cells. In summary, these findings suggest that Capsanthin effectively inhibits the migration and metastatic capabilities of the highly invasive 231Br cell line by downregulating EZH2 and EMT markers at both the RNA and protein levels.

### Capsanthin inhibits migration and reduces EZH2 and EMT marker expression in mesenchymal stem cells (3A6)

**Fig. 4:**
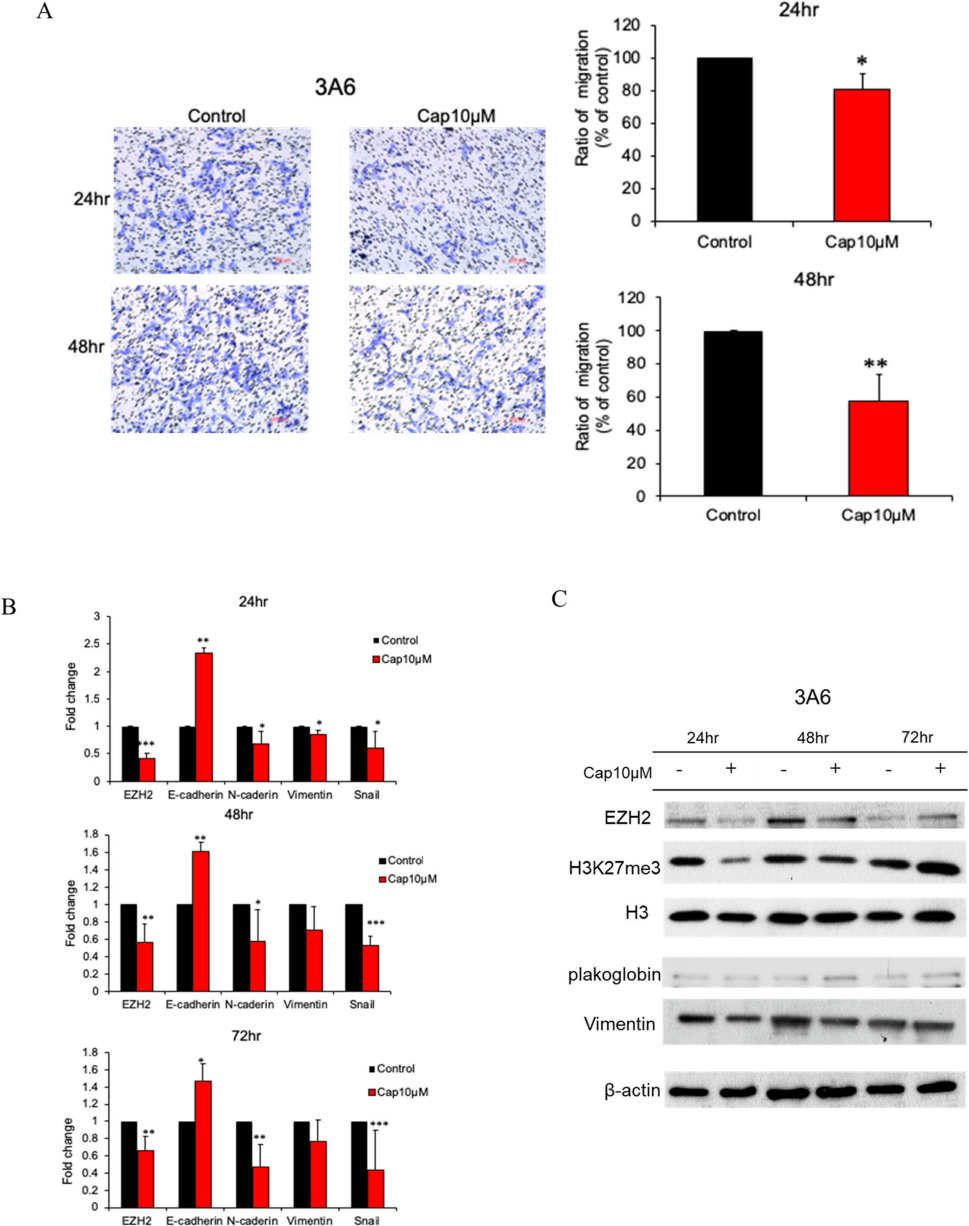
Capsanthin inhibits migration of mesenchymal stem cells (3A6). **A** Transwell migration assays demonstrate that Capsanthin treatment (10 μM) significantly reduces the migration of 3A6 mesenchymal stem cells at 24 and 48 h. Student’s *t*-test (*n* = 3). **B** qRT-PCR analysis shows that Capsanthin treatment reduces the mRNA expression of EZH2 and EMT markers in 3A6 cells at 24, 48, and 72 h. Student’s *t*-test (*n* = 3). **C** Western blot analysis indicates that Capsanthin treatment reduces the protein levels of EZH2 and EMT markers in 3A6 cells at 24, 48, and 72 h. (**p < 0.01, ***p < 0.001).

Mesenchymal stem cells (MSCs) play a crucial role in tumor progression and metastasis. They have been shown to migrate to tumor sites, where they promote cancer cell growth, angiogenesis, and metastasis by secreting various cytokines and growth factors. MSCs can also contribute to the formation of a pro-tumorigenic environment, facilitating cancer cell invasion and metastasis through the EMT process [18–20]. Our results from a transwell migration assay demonstrate that Capsanthin significantly inhibits the migration ability of mesenchymal stem cells (3A6). After 24 h of treatment with 10 μM Capsanthin, there was a noticeable reduction in cell migration compared to the control group. This inhibitory effect was even more pronounced after 48 h of treatment (Fig. 4A). The results indicated that Capsanthin also impacts the expression of EZH2 and EMT markers in 3A6 cells. Capsanthin treatment led to a significant downregulation of EZH2, E-cadherin, N-cadherin, Vimentin, and Snail mRNA levels at 24, 48, and 72 h. These findings suggest that Capsanthin effectively inhibits key regulators of EMT in these cells (Fig. 4B). Further supporting this, our Western blot analysis shows that Capsanthin treatment reduced the protein levels of EZH2, H3K27me3, plakoglobin, and Vimentin in 3A6 cells at 24, 48, and 72 h. This confirms the suppression of EMT markers at the protein level, indicating that Capsanthin not only reduces the expression of EZH2 but also inhibits the EMT process in mesenchymal stem cells (Fig. 4C). Overall, these results indicate that Capsanthin effectively inhibits the migration and metastatic capabilities of mesenchymal stem cells by downregulating EZH2 and EMT markers at both the RNA and protein levels.

### Capsanthin inhibits the enhanced migration ability of MSCs (3A6) co-cultured with TNBC cells or brain metastatic TNBC cells

**Fig. 5:**
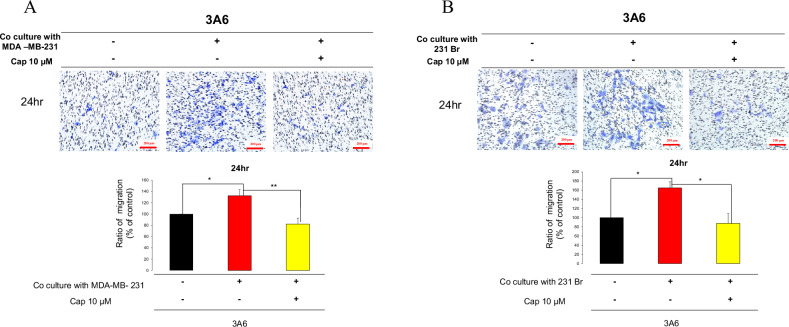
Mesenchymal stem cell co-culture increases migration ability, inhibited by Capsanthin. **A** Co-culturing mesenchymal stem cells (3A6) with MDA-MB-231 breast cancer cells significantly increases the migration ability of 3A6 cells. Capsanthin treatment (10 μM) effectively inhibits this enhanced migration at 24 h. Student’s *t*-test (*n* = 3). **B** Co-culturing 3A6 cells with the brain metastatic breast cancer cell line 231Br increases the migration of 3A6 cells, which is significantly reduced by Capsanthin treatment (10 μM) at 24 h. Student’s *t*-test (*n* = 3). (*p < 0.05, **p < 0.01).

Due to our preliminary findings that Capsanthin can inhibit the migration ability of 231Br and 3A6 cells, we further evaluated the effects using a co-culture system with 231Br and 3A6 cells. The results indicate that co-culturing mesenchymal stem cells (3A6) with MDA-MB-231 breast cancer cells significantly increases the migration ability of 3A6 cells. However, treatment with 10 μM Capsanthin effectively inhibits this enhanced migration ability. After 24 h of Capsanthin treatment, there was a noticeable reduction in cell migration compared to the co-culture control group without Capsanthin. This inhibitory effect was even more pronounced after 48 h of treatment (Fig. 5A). Similarly, when 3A6 cells were co-cultured with the brain metastatic breast cancer cell line 231Br, an increase in the migration ability of 3A6 cells was observed. Treatment with 10 μM Capsanthin significantly inhibited this enhanced migration. The reduction in cell migration was evident after 24 h of Capsanthin treatment and further confirmed after 48 h (Fig. 5B). These findings suggest that Capsanthin can effectively counteract the increased metastatic potential conferred by the presence of breast cancer cells or brain metastatic breast cancer cells on mesenchymal stem cells. The ability of Capsanthin is to inhibit the migration of 3A6 cells suggests its potential as a therapeutic agent in preventing the supportive role of MSCs in tumor metastasis.

### KEGG pathway enrichment analysis of differentially expressed genes in patient samples

**Fig. 6:**
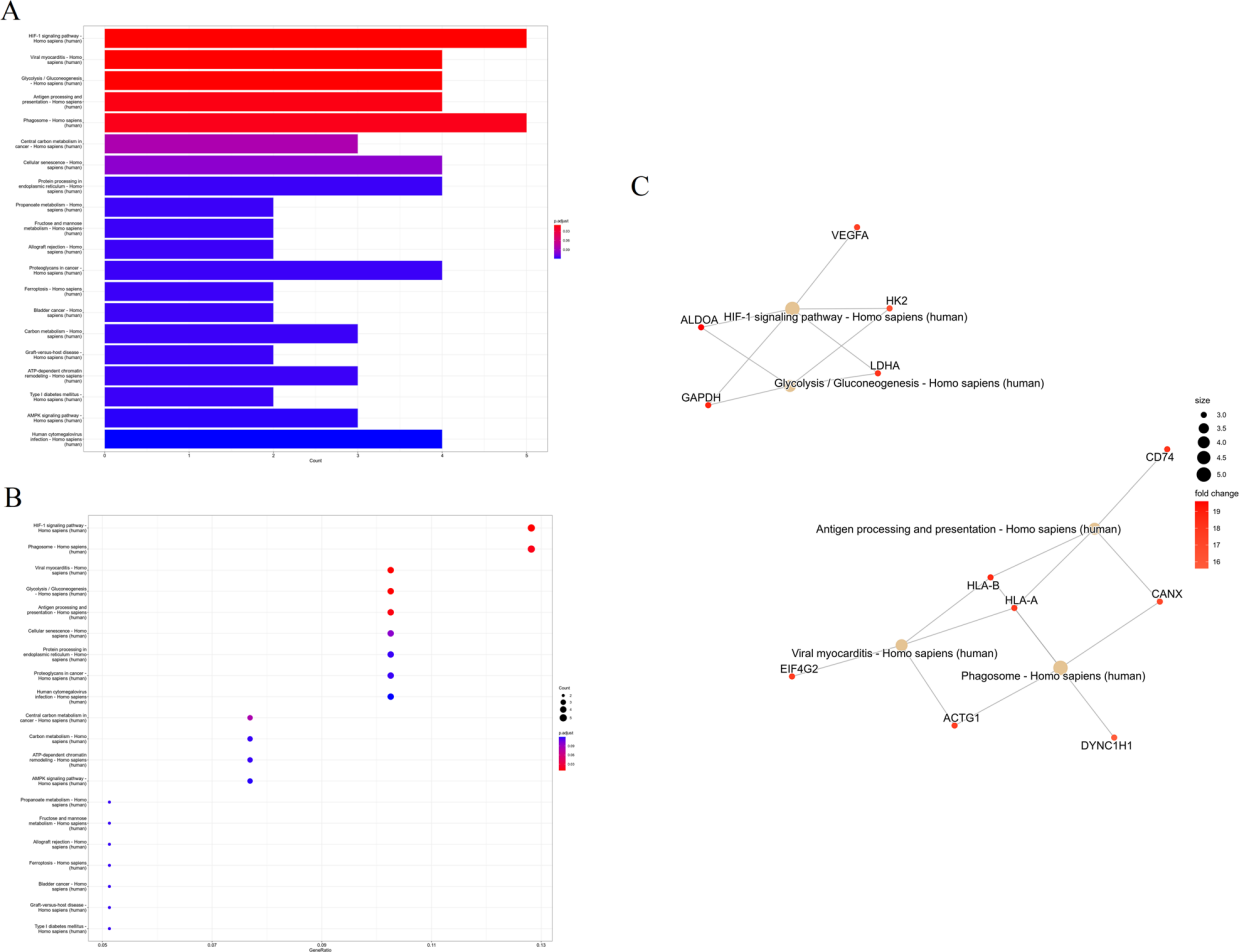
KEGG pathway enrichment analysis. **A** Bar plot showing significant pathways associated with differentially expressed genes between primary breast cancer and brain metastatic samples. Key pathways include HIF-1 signaling, glycolysis/gluconeogenesis, and antigen processing and presentation. **B** Dot plot indicating the significance of these pathways, with gene ratios and adjusted *p*-values. **C** Network plot illustrating the connections between key genes and their associated pathways, highlighting the roles of metabolic reprogramming and immune evasion in brain metastasis.

Using RNA-sequencing analysis, we examined samples from three patients with primary breast cancer and their corresponding brain metastases. The KEGG pathway enrichment analysis revealed several significant pathways associated with differentially expressed genes between the primary breast cancer samples and the brain metastatic samples. The bar plot (Fig. 6A) highlights the enriched pathways, including the HIF-1 signaling pathway, glycolysis/gluconeogenesis, and antigen processing and presentation among the most significant pathways identified. Other notable pathways include phagosome, cellular senescence, and protein processing in the endoplasmic reticulum. The dot plot (Fig. 6B) further validates the significance of these pathways, with the HIF-1 signaling pathway and glycolysis/gluconeogenesis showing the highest gene ratios and lowest adjusted p-values, indicating a strong association with the differentially expressed genes. The network plot (Fig. 6C) demonstrates the connections between key genes and their associated pathways. For instance, VEGFA, HK2, and LDHA are linked to the HIF-1 signaling pathway and glycolysis/gluconeogenesis, underscoring their roles in the metabolic adaptations of the metastatic brain cancer cells. Additionally, CD74 is associated with antigen processing and presentation, suggesting an immune-related component in the brain metastases. Overall, these analyses suggest that metabolic reprogramming and immune evasion are prominent features in the brain metastatic samples compared to the primary breast cancer samples.

### Evaluation of immune checkpoint expression between MDA-MB-231 and 231Br cells

**Fig. 7:**
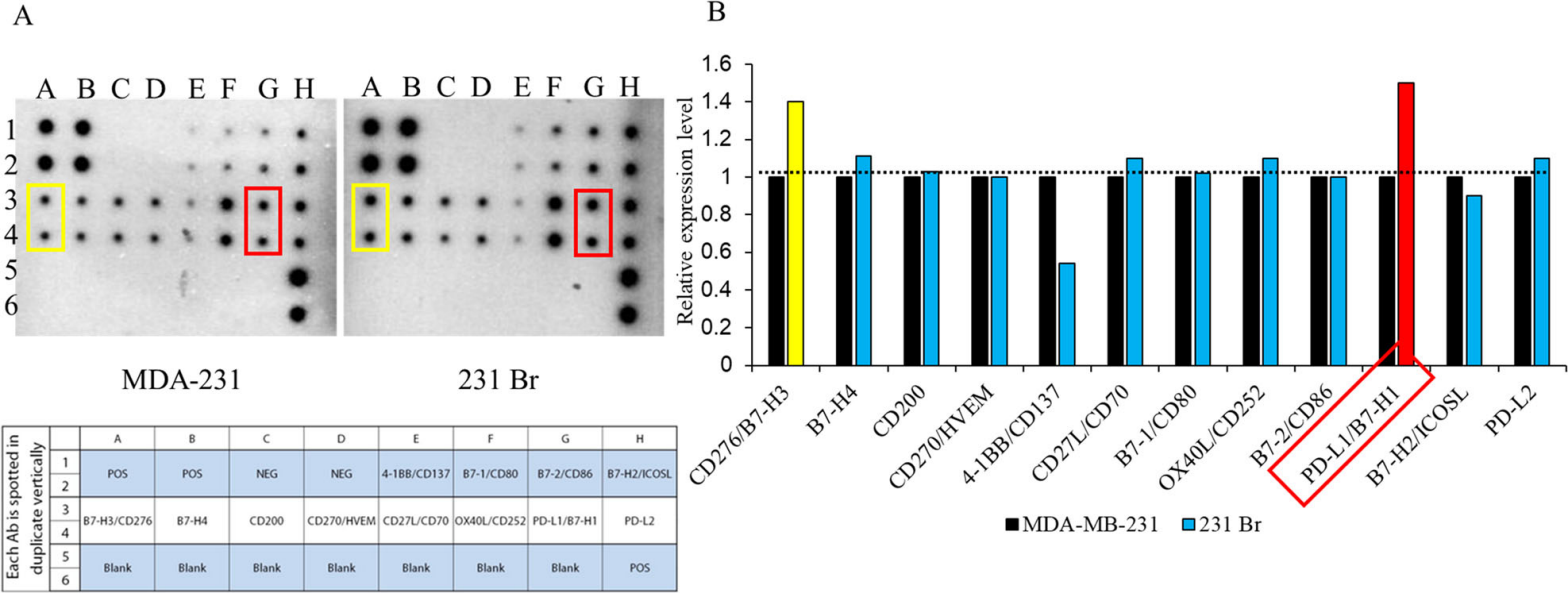
Expression of immune checkpoints in MDA-MB-231 and 231Br cells. **A** Immune checkpoint array analysis showing the expression levels of various immune checkpoints, including PD-L1, in MDA-MB-231 and 231Br cells. PD-L1 expression is significantly higher in 231Br cells. **B** Quantitative analysis confirms the increased expression of immune checkpoints, particularly PD-L1, in 231Br cells compared to MDA-MB-231 cells.

Our results reveal differential expression of immune checkpoints between the primary breast cancer cell line MDA-MB-231 and its brain metastatic counterpart 231Br. Using an immune checkpoint array, we observed notable differences in the expression levels of several key immune checkpoints. The array analysis demonstrated that PD-L1 (B7-H1) expression was significantly higher in 231Br cells compared to MDA-MB-231 cells (Fig. 7A). Additionally, we observed elevated levels of B7-1 (CD80), B7-2 (CD86), and B7-H2 (ICOSL) in 231Br cells, indicating an increased expression of these immune checkpoints in the brain metastatic cells (Fig. 7A). Quantitative analysis further confirmed these findings, showing a relative increase in PD-L1, B7-1, B7-2, and B7-H2 expression levels in 231Br cells compared to MDA-MB-231 cells (Fig. 7B). The elevated expression of these immune checkpoints suggests a potential mechanism by which 231Br cells evade immune surveillance and contribute to the metastatic progression in the brain. Overall, these findings highlight the differential immune checkpoint expression profiles between primary and brain metastatic breast cancer cells, with significant upregulation of PD-L1 and other key immune checkpoints in 231Br cells, which may play a crucial role in their enhanced metastatic potential and immune evasion capabilities.

### The interaction between EZH2 and PD-L1 in TNBC and 231Br cells

**Fig. 8:**
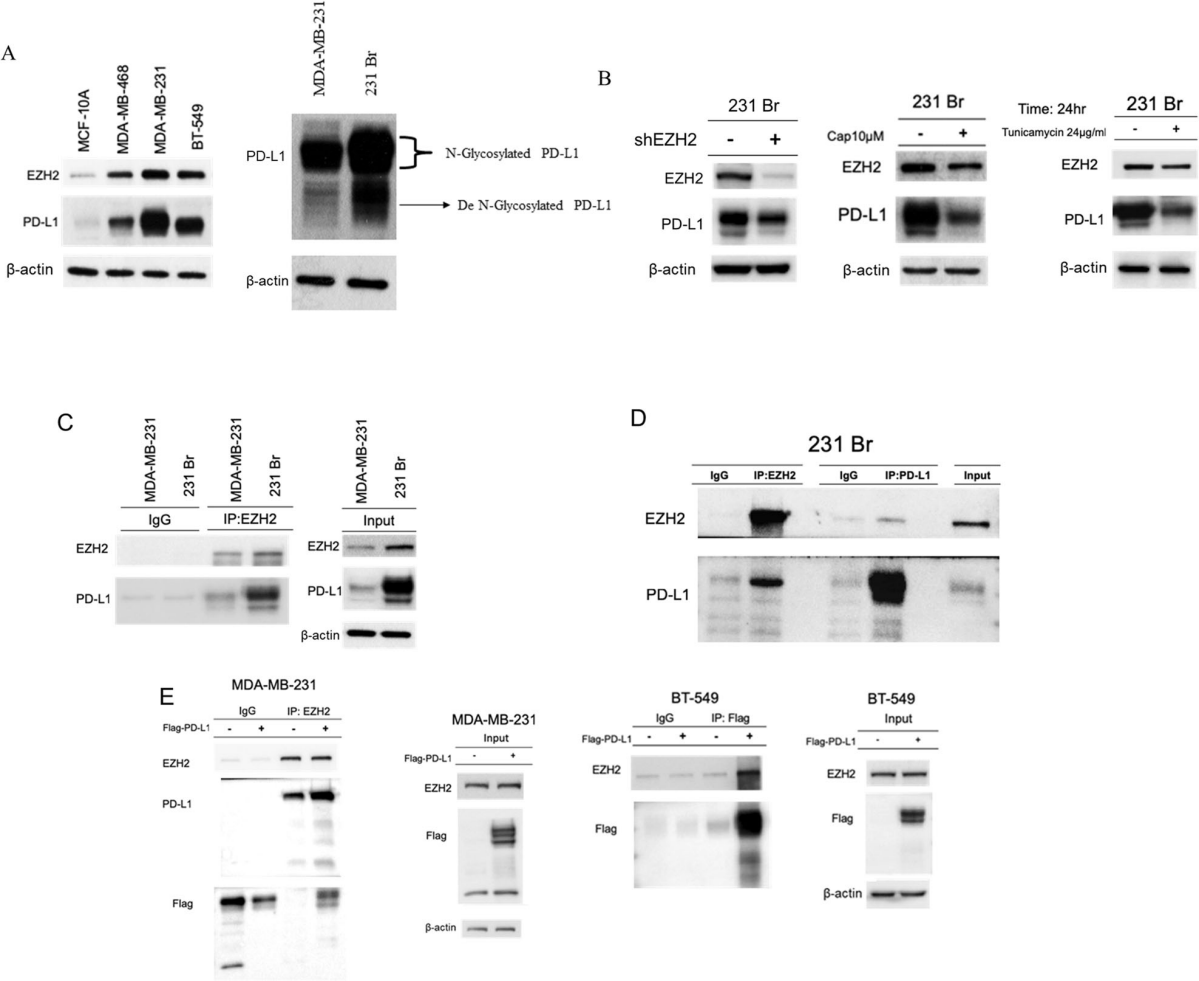
The interaction between EZH2 and PD-L1 in TNBC and MDA-231Br cells. **A** Western blot analysis showing the expression of EZH2 and PD-L1 across different breast cancer cell lines (MCF-10A, MDA-MB-468, MDA-MB-231, BT-549) and the brain metastatic variant 231Br. The results indicate higher levels of both EZH2 and PD-L1 in the TNBC cell lines compared to the non-tumorigenic MCF-10A cell line. Notably, 231Br cells exhibit increased N-glycosylated PD-L1 compared to MDA-MB-231 cells, indicated by the distinct bands corresponding to the glycosylated and de-glycosylated forms of PD-L1. **B** Western blot analysis of 231Br cells showing the effects of EZH2 knockdown (shEZH2), Capsanthin and Tunicamycin treatment on PD-L1 expression. Both interventions result in a reduction of N-glycosylated PD-L1 levels and overall PD-L1 expression, suggesting that EZH2 positively regulates PD-L1 glycosylation. **C** Immunoprecipitation (IP) assay demonstrated the interaction between EZH2 and PD-L1 in MDA-MB-231 and 231Br cells. Immunoprecipitation with anti-EZH2 antibodies pulls down PD-L1, confirming the physical interaction between these proteins in both cell lines. **D** Western blot analysis of immunoprecipitates from 231Br cells showing that EZH2 and PD-L1 interact directly, confirming the association between these two proteins in the context of brain metastasis in TNBC. **E** Western blot analysis of MDA-MB-231 and BT-549 cells transfected with Flag-tagged PD-L1. The transfection increases the levels of N-glycosylated PD-L1 and enhances the interaction between EZH2 and PD-L1, as shown by co-immunoprecipitation.

Our previous results indicated that PD-L1 expression is higher in brain metastatic samples compared to primary breast cancer samples. Previous studies have shown that HIF-1α can enhance the expression of PD-L1, facilitating cancer cell survival under hypoxic conditions. Additionally, N-linked glycosylated PD-L1 has been implicated in cancer metastasis, further highlighting the significance of PD-L1 regulation in cancer progression [21, 22]. In this study, we further investigated the interaction between EZH2 and PD-L1 in TNBC and brain metastatic 231Br cells. The expression levels of EZH2 and PD-L1 were analyzed across different TNBC cell lines (MDA-MB-468, MDA-MB-231, and BT-549) and compared with 231Br cells. We observed that both EZH2 and PD-L1 are highly expressed in the MDA-MB-231 and BT-549 cells, with an even higher expression in the brain metastatic 231Br cells (Fig. 8A). The results also showed that in 231Br cells, PD-L1 exists in both its N-glycosylated and de-N-glycosylated forms (Fig. 8A). Knockdown of EZH2 in 231Br cells significantly reduced PD-L1 expression, while treatment with Capsanthin also led to a decrease in PD-L1 levels. Additionally, we utilized Tunicamycin, a glycosylation inhibitor, to examine its effects on N-glycosylated PD-L1 in 231Br cells. As shown in the results, Tunicamycin significantly inhibits N-glycosylated PD-L1 expression in 231Br cells without affecting EZH2 levels (Fig. 8B). These findings suggest a potential new combination therapy approach for treating breast cancer brain metastases. Immunoprecipitation experiments demonstrated that EZH2 interacts directly with PD-L1 in both MDA-MB-231 and 231Br cells, as evidenced by the presence of PD-L1 in the EZH2 immunoprecipitated complex (Fig. 8C, D). Furthermore, the transfected with Flag-tagged PD-L1 in MDA-MB-231 and BT-549 cells not only increased the expression of N-glycosylated PD-L1 but also confirmed the interaction between EZH2 and PD-L1 (Fig. 8E). Overall, these findings suggest that EZH2 upregulates PD-L1 expression and promotes its N-linked glycosylation, contributing to the metastatic potential of TNBC cells, particularly in brain metastasis. These interactions highlight the importance of targeting the EZH2-PD-L1 axis for therapeutic interventions in metastatic TNBC.

### Capsanthin decreased the expression of EZH2 and PD-L1 in 231Br cell-derived tumors in mice

**Fig. 9:**
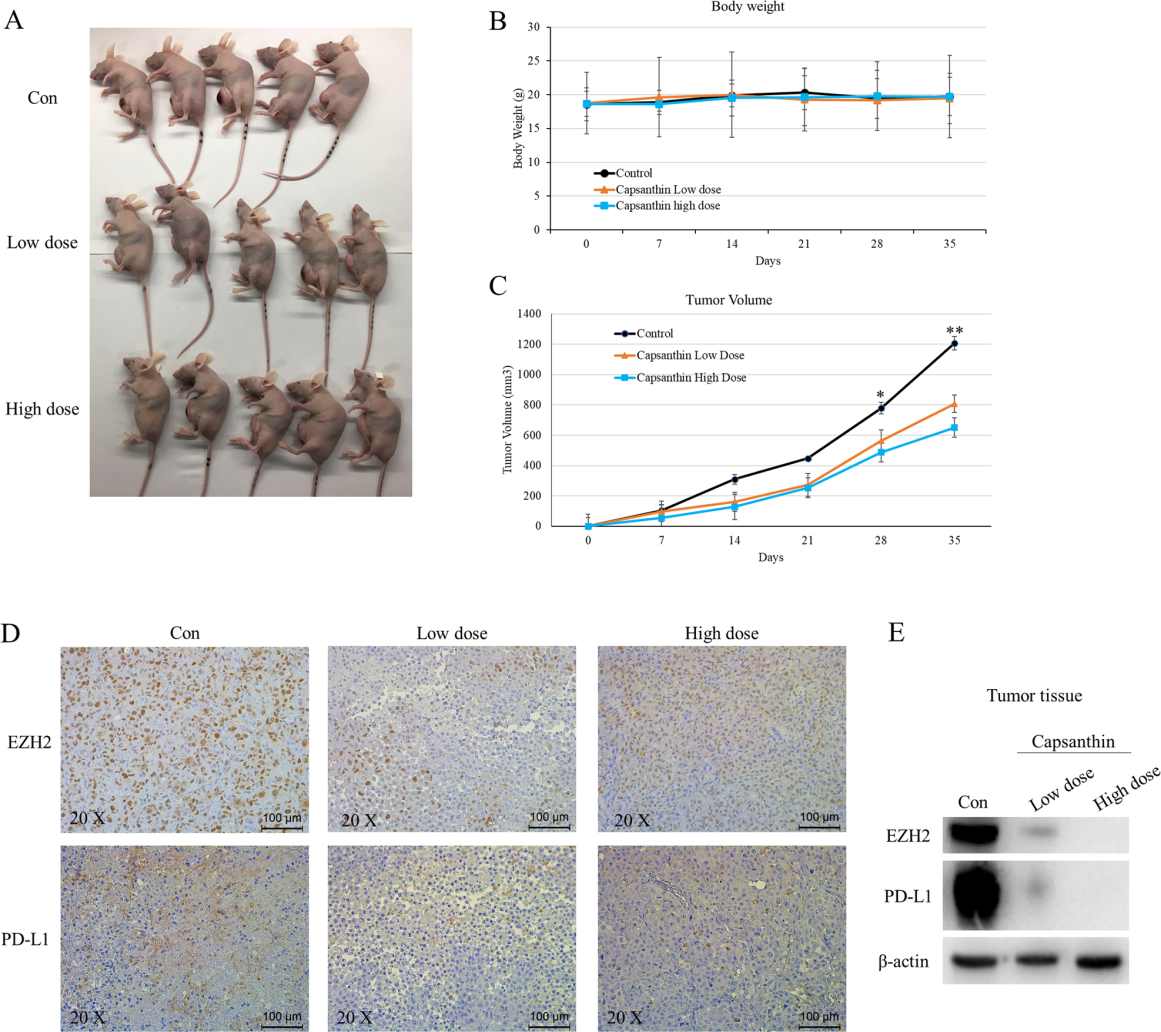
Capsanthin decreases the expression of EZH2 and PD-L1 in 231Br cell-derived tumors in mice. **A** Experimental design: Nude mice were injected with 231Br cells into the mammary fat pad and randomly divided into three groups (control group: PBS; low-dose Capsanthin: 0.036 mg/kg; high-dose Capsanthin: 0.36 mg/kg). Treatments were administered intraperitoneally twice a week for 14 days. **B** Body weights of the mice were measured throughout the study and showed no significant differences among the groups, indicating that Capsanthin does not adversely affect normal physiological functions. Student’s t-test (*n* = 3). **C** Tumor volumes were significantly reduced in Capsanthin-treated groups, with a pronounced inhibitory effect observed after the third week of treatment. Student’s *t*-test (*n* = 3). **D** Immunohistochemistry (IHC) analysis revealed decreased EZH2 and PD-L1 expression levels in tumors from Capsanthin-treated groups compared to controls. **E** Western blot analysis further confirmed the reduction of EZH2 and N-glycosylated PD-L1 expression in Capsanthin-treated tumors. (*p < 0.05, **p < 0.01).

To further validate whether Capsanthin can influence the expression of EZH2 and PD-L1 in vivo, we injected 231Br cells into the mammary fat pad of nude mice. Next, the nude mice were randomly divided into three groups (5 mice per group): the control group (injected with PBS), the low-dose Capsanthin group, and the high-dose Capsanthin group. Over a 14-day period, the body weight and tumor size of the mice were measured. At the end of the 14 days, the mice were sacrificed (Fig. 9A), and the tumors were harvested for immunohistochemistry (IHC). Additionally, the tumor tissues were homogenized, and the expression levels of EZH2 and PD-L1 were analyzed using Western blotting. The results clearly show that Capsanthin administration does not significantly impact the body weight of nude mice throughout the study period (Fig. 9B). This observation suggests that Capsanthin may have minimal or negligible effects on normal physiological functions, indicating its potential safety and tolerability for in vivo applications. In contrast, a marked tumor-suppressive effect was observed, with significant inhibition of tumor growth becoming evident after the third week of treatment (Fig. 9C). This highlights the potential therapeutic benefits of Capsanthin in reducing tumor progression without compromising the general health of the animals. Additionally, both IHC and Western blot analysis provided strong evidence that Capsanthin effectively downregulates the expression of EZH2 and N-glycosylated PD-L1 in tumor cells within the animal model (Fig. 9D, E). These results suggest that mechanism of Capsanthin extends to in vivo systems, where it can modulate key molecular pathways associated with tumor cell proliferation and immune evasion. By targeting the EZH2-PD-L1 axis, Capsanthin not only inhibits tumor progression but also potentially enhances anti-tumor immune responses. These findings further underscore the therapeutic potential of Capsanthin as a safe and effective agent for combating aggressive tumors, such as those in TNBC brain metastases.

## Discussion

**Fig. 10:**
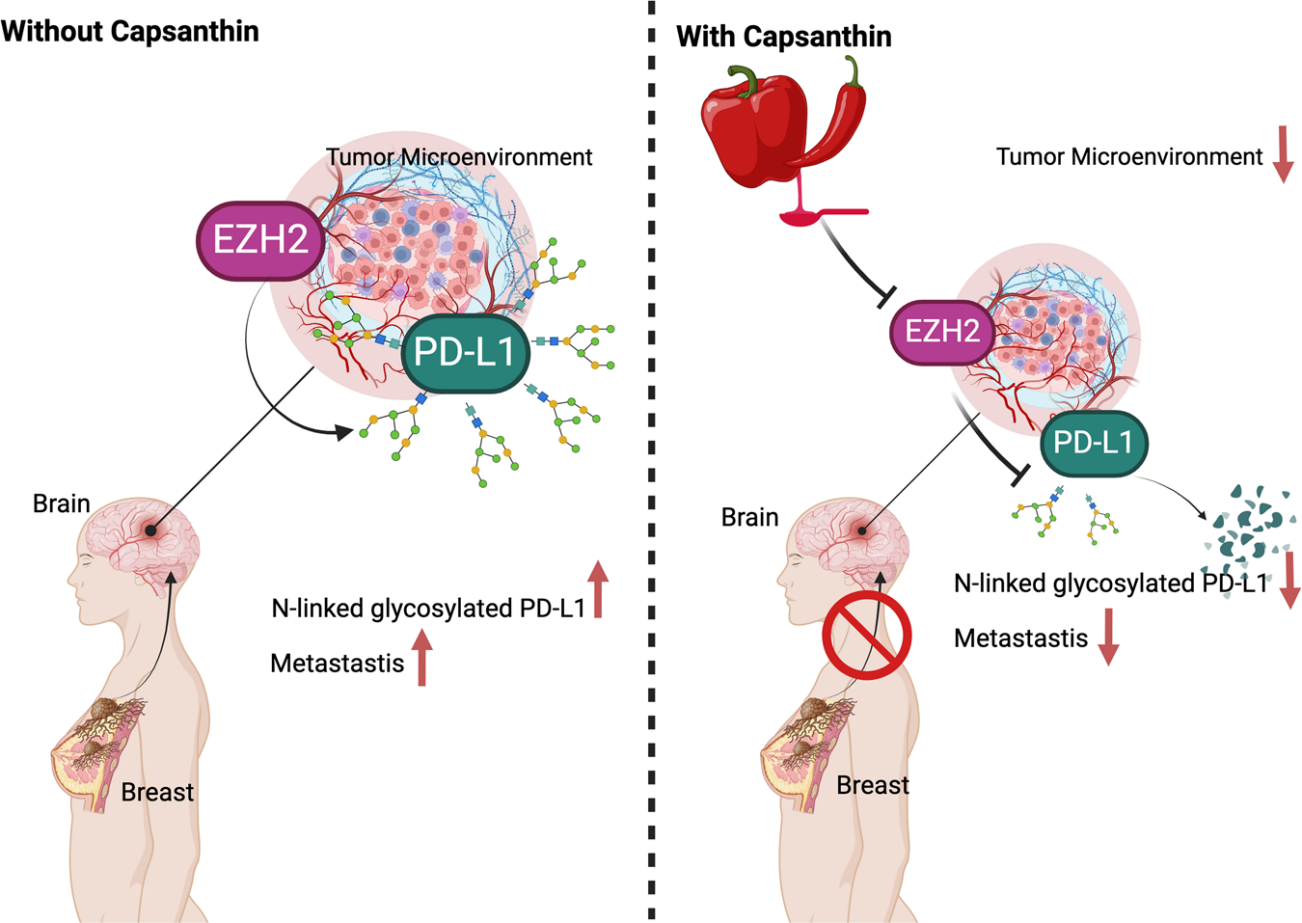
A proposed illustration for the molecular mechanism of EZH2 and PD-L1 interaction and modulation by Capsanthin in TNBC and 231Br cells.

Our comprehensive study investigates the intricate dynamics between EZH2 and PD-L1 in TNBC and its brain metastatic variant, 231Br cells. Our findings align with existing literature and provide deeper insights into the epigenetic regulation, immune escape mechanisms, and metastatic progression of TNBC. EZH2, a critical epigenetic regulator, is frequently overexpressed in various cancers, including breast, prostate, lung, hepatocellular, and colorectal cancers [16, 23]. It facilitates tumor progression by trimethylating histone H3 at lysine 27 (H3K27me3), leading to transcriptional repression of tumor suppressor genes [16]. In our study, high levels of EZH2 in brain metastatic 231Br cells correlated with increased PD-L1 expression, promoting immune evasion (Fig. 1A). The overexpression of EZH2 and its association with poor prognosis have been extensively documented. For example, Varambally et al. highlighted the association between EZH2 and poor prognosis in prostate cancer [17]. Furthermore, the upregulation of EZH2 has been linked to advanced stages of lung, glioblastoma, and colorectal cancers, confirming its role in cancer aggressiveness and metastasis [24–26]. PD-L1 is a pivotal immune checkpoint protein that enables cancer cells to evade immune surveillance by inhibiting T cell activation. This mechanism is particularly relevant in TNBC, known for its high invasiveness and resistance to conventional therapies [27, 28]. Our study demonstrated significantly higher PD-L1 expression in brain metastatic 231Br cells compared to primary MDA-MB-231 cells, aligning with literature that hypoxia-induced PD-L1 expression facilitates immune escape [28]. Glycosylation, especially N-linked glycosylation, is crucial for the stability and function of PD-L1. Studies indicate that glycosylated PD-L1 is more effective in mediating immune escape and promoting metastasis [29–32]. Several natural compounds, including curcumin and resveratrol, have demonstrated promising immune-modulatory effects in cancer by targeting immune checkpoint pathways and reducing PD-L1 expression, which enhances anti-tumor immune responses [3, 15]. The impact of Capsanthin on the EZH2-PD-L1 axis aligns with these findings, suggesting it could similarly complement immune checkpoint inhibitors. By inhibiting EZH2, Capsanthin reduces PD-L1 stability and expression, effectively restoring immune detection capabilities. This parallel with other natural compounds reinforces the potential for Capsanthin as an adjunct therapy, particularly in TNBC patients with brain metastasis. Recent studies indicate that EZH2, through its interaction with pathways such as STAT3, contributes to immune evasion by upregulating PD-L1 expression, which facilitates tumor progression [21, 22]. For example, EZH2 has been shown to influence PD-L1 transcription via miR-338-3p and other modulators, thereby enhancing immune evasion capabilities. The inhibitory effect of Capsanthin on EZH2 observed in our study suggests it may interrupt these pathways, potentially sensitizing tumor cells to immune responses. Our results support the findings that inhibiting EZH2, either through Capsanthin treatment or EZH2 shRNA in 231Br cells, effectively reduces N-glycosylated PD-L1 levels and decreases the interaction between EZH2 and N-glycosylated PD-L1. Similarly, transfection of MDA-MB-231 and BT549 cells with Flag-tagged PD-L1 leads to an increase in N-glycosylated PD-L1 expression and enhances the interaction between EZH2 and N-glycosylated PD-L1. (Fig. 8). This underscores the importance of post-translational modifications in PD-L1-mediated metastatic processes. The results of our study suggest that targeting the EZH2-PD-L1 axis could be a promising therapeutic strategy in TNBC. Inhibitors of EZH2 have shown potential in reducing tumor growth and enhancing the efficacy of immune checkpoint inhibitors (ICIs) like anti-PD-1/PD-L1 antibodies in various cancers [23]. Combination therapies including EZH2 inhibitors could improve the response rates and durability of benefits in patients with metastatic TNBC. Additionally, the role of glycosylation in PD-L1 functionality has been extensively documented. Glycosylation inhibitors like Tunicamycin have been shown to reduce PD-L1 levels and enhance the effectiveness of ICIs, mirroring our findings in 231Br cells. In conclusion, our study underscores the effect of Capsanthin and the pivotal roles of EZH2 and PD-L1 in TNBC progression and metastasis (Fig. 10). The findings highlight the potential therapeutic value of targeting the EZH2-PD-L1 axis in metastatic TNBC. These insights contribute to the broader understanding of cancer biology and suggest new avenues for therapeutic intervention. Further research and clinical trials are essential to validate these strategies and improve outcomes for patients with metastatic breast cancer.

## Materials and methods

### Clinical samples

Paired tissue samples from primary breast cancer and brain metastases (*n* = 3) were collected from patients at China Medical University Hospital (CMUH111-REC1-023). All specimens obtained informed consent from patients and were approved by the hospital’s ethics committee.

### Cell lines, cell culture conditions, and Capsanthin stock solution

The TNBC brain metastasis cell line 231Br was generously provided by Dr. Dihua Yu from the University of Texas MD Anderson Cancer Center [33–35]. Other cell lines used in this study were sourced from the American Type Culture Collection. Human TNBC cell lines (MDA-MB-231, MDA-MB-468, and 231Br) were cultured in DMEM/F12 (1:1) supplemented with 10% (v/v) fetal bovine serum (Gibco). The human TNBC cell lines BT-549 and BT-20 were maintained in RPMI-1640 medium supplemented with 10% (v/v) fetal bovine serum and 1% (w/v) penicillin/streptomycin. The human mesenchymal stem cell 3A6 was cultured in DMEM (1:1) supplemented with 10% (v/v) fetal bovine serum (Gibco). The non-tumorigenic mammary epithelial cell line MCF-10A was cultured in DMEM/F12 medium supplemented with 1.05 mM CaCl_2_, 100 ng/mL cholera toxin, 5% (v/v) horse serum (Gibco), 10 μg/mL insulin, 100 U/mL penicillin, 100 μg/mL streptomycin, 20 ng/mL EGF (Sigma), and 500 ng/mL hydrocortisone. All cell lines were maintained in a humidified incubator at 37 °C with 5% CO_2_. Capsanthin was dissolved in DMSO at a stock concentration of 60 mM and stored at −20 °C.

### Comprehensive analyses of EZH2 from TCGA

UALCAN is an interactive and user-friendly web platform designed for cancer omics data analysis. It utilizes RNA-sequencing data and clinical information from 31 cancer types provided by TCGA [36]. Gene Expression Profile Interactive Analysis 2 (GEPIA2), is an updated tool for RNA expression analysis, incorporating data from 9,736 tumor samples and 8,587 normal samples from TCGA and GTEx projects, all processed through a standardized pipeline [37]. We used both UALCAN and GEPIA2 to identify differentially expressed genes between tumor and normal tissues and to analyze overall patient survival in relation to EZH2 expression levels in breast cancer patients.

### Transwell cell migration assays

TNBC cells and 3A6 cells (2 × 10^4^ cells per well) were suspended in 100 μL of serum-free medium with or without 10 μM Capsanthin and then added to the upper chamber filter inserts (Millipore). Next, 700 μL of DMEM/F12 (1:1) with 10% fetal bovine serum was added to the lower chamber to act as a chemoattractant. The chambers were incubated at 37 °C for 24 h. Cells that migrated and adhered to the lower surface of the filter were fixed and stained for 15–20 min using a solution of 37% formaldehyde in ethanol containing 1% (w/v) crystal violet. The stained cells that invaded or migrated through the membrane were counted using a light microscope at 2.5× and 10× magnification. To quantify the staining, crystal violet was dissolved in 33% acetic acid, and absorbance was measured at 595 nm.

### IncuCyte assays

MDA-MB-231, BT-549, and MDA-MB-468 cells (1 × 10^5^ cells per well) were seeded into a 96-well culture plate and incubated at 37 °C for 24 h. Wounds were created using a 96-well WoundMaker (Essen BioScience). The cells were then washed twice with phosphate-buffered saline (PBS) and either left untreated or treated with 10 μM Capsanthin. Images of the wounds were captured automatically within the CO_2_ incubator using IncuCyte software (Essen BioScience). These images were taken at 4-hour intervals throughout the 24-hour experiment. Data were analyzed based on wound confluence, with values calculated using the IncuCyte software package (Essen BioScience).

### Western blotting

To generate whole-cell lysates, cells were washed twice with ice-cold PBS and then lysed by sonication in NETN buffer (20 mM Tris, pH 8.0, 150 mM NaCl, 1 mM EDTA, pH 8.0, 0.5% (v/v) Nonidet P-40), supplemented with protease and phosphatase inhibitors (25 mM NaF, 2 mM Na_3_VO_4_, 0.1 mM phenylmethylsulfonyl fluoride, 20 μg/ml aprotinin). Protein samples (10–30 μg) were loaded onto a 10% or 12% SDS-PAGE gel for separation, followed by transfer onto a polyvinylidene difluoride membrane. Primary antibodies used included mouse monoclonal antibodies against vimentin (Cell Signaling, #5741) and β-actin (GeneTex, #GTX109639), and plakoglobin (Abcam, #EPR17310); rabbit monoclonal antibodies against PD-L1 (GeneTex, #GTX636033), E-cadherin (Cell Signaling, #3195), EZH2 (Cell signaling, #5246), H3K27me3 (Abcam, #ab6002), and H3 (Abcam, #ab18521); and rabbit polyclonal antibodies against N-cadherin and Flag (GeneTex, #GTX115043). Membranes were incubated overnight at 4 °C with primary antibodies, followed by washing with PBS containing 0.1% (w/v) Tween 20. They were then incubated for 1 h at room temperature with horseradish peroxidase-conjugated secondary antibodies (Gentex, #GTX213110-01 and #GTX213111-01). Immunoreactive bands were visualized using an enhanced chemiluminescence detection reagent (GE, Piscataway, NJ).

### Gene knockdown via shRNA

Gene knockdown was performed using specific shRNAs delivered via the lentiviral system provided by the National RNAi Core Facility (Academia Sinica, Taipei, Taiwan), following their instruction manual. The shRNA construct targeting EZH2 was clone TRC0000040076. As a negative control, the shRNA construct against luciferase (shLuc), clone TRCN0000072244, was used. The efficiency and specificity of the gene knockdown were verified according to previously published protocols [38].

### Plasmids and transient transfection

The Flag-PD-L1 plasmid was obtained from Addgene (Cambridge, MA). Transfections were carried out using Lipofectamine 2000 according to the manufacturer’s instructions. After 48 h, the transfected cells were used for subsequent experiments [39].

### Quantitative reverse transcription-PCR and quantitative real-time PCR

Total RNA was extracted using TRIzol reagent (Invitrogen) following the manufacturer’s instructions. All the primers (Table 1) were synthesized by Invitrogen. Quantitative PCR (qPCR) was performed as previously described [38, 40], and detected using the LightCycler 480 system (Roche).

**Table 1:** The primer sequence of genes for QPCR.

Gene	Sequence
EZH2 forward	CAGTAAAAATGTGTCCTGCAAGAA
EZH2 reverse	TCAAGGGATTTCCATTTCTCTTTCGA
E-cadherine forward	GGAACTATGAAAAGTGGGCTTG
E-cadherine reverse	AAATTGCCAGGCTCAATGAC
N-cadherine forward	GGTGGAGGAGAAGAAGACCAG
N-cadherine reverse	GGCATCAGGCTCCACAGT
Vimentin forward	TCTCTGAGGCTGCCAACCG
Vimentin reverse	CGAAGGTGACGAGCCATTTCC
Snail forward	CTTCCAGCAGCCCTACGAC
Snail reverse	CGGTGGGGTTGAGGATCT
GAPDH forward	CCACCCATGGCAAATTCCATGGCA
GAPDH reverse	TCTAGACGGCAGGTCAGGTCCACC

### Human immune-checkpoint array

We employed the Ray Biotech (C-series ISO 13485) assay for our analysis. The concentration of each cell lysate was empirically adjusted to ensure optimal sensitivity while minimizing background noise. Cells cultured in DMEM/F12 with 10% fetal bovine serum were trypsinized, rinsed with PBS, and the cell lysate concentration was set to 1 mg/ml. Following this, we adhered to all reagent preparation and procedural steps outlined in the Ray Biotech protocol. Each membrane was subsequently exposed to X-ray film for 1–5 min, with multiple exposure times recommended by Ray Biotech. An array map was generated and utilized for comparative analysis.

### Immunoprecipitation assay

TNBC cells were lysed using an immunoprecipitation lysis buffer containing 50 mM Tris-Cl (pH 7.5), 150 mM NaCl, and 1% (v/v) Triton X-100, along with protease and phosphatase inhibitors. Equal amounts of protein were then incubated with the appropriate antibody and Protein A/G agarose (Thermo Fisher, 20421) at 4 °C for 16 h. The beads were washed five times with the immunoprecipitation lysis buffer, and the bound proteins were eluted with 2× sample loading dye. The eluted proteins were then subjected to SDS-PAGE, followed by detection through western blotting.

### RNA-sequencing analysis

mRNA samples from three pairs of primary and brain metastasis TNBC patients were sent to Welgene Biotech (Taipei, Taiwan) for RNA-sequencing analysis. Differential gene expression analysis was performed using the Bioconductor package Limma [41]. Genes with differential expression were identified based on fold-change thresholds of ≥1.5 or ≤0.7 and a false discovery rate (FDR) of <0.1, using a modified t-test. To explore the pathways associated with these differentially expressed genes in brain metastases, gene set enrichment analysis (GSEA) was conducted, identifying the top 10 significant pathways with an FDR of <0.25 [42].

### Tumor orthotopic model

Four-week-old female BALB/c nude mice were obtained from the Laboratory Animal Center, College of Medicine, National Taiwan University, Taipei, Taiwan. The mice were housed in a sterile, pathogen-free environment and maintained according to the Institutional Animal Ethical Guidelines of China Medical University (assignment number: CMUIACUC-2020-039). To ensure unbiased allocation of animals to experimental groups, a randomization process was employed prior to cell inoculation. Specifically, a computer-generated random number sequence was used to assign each mouse to one of the three experimental groups: control (PBS), low-dose Capsanthin (0.036 mg/kg), or high-dose Capsanthin (0.36 mg/kg). This approach minimized selection bias and ensured a balanced distribution of mice across the groups. Additionally, all sample analyses were conducted in a blinded manner to further prevent any potential bias during data collection and interpretation. Each mouse was orthotopically injected with 5 × 10^5^ tumor cells into the mammary fat pad. We included a minimum of five animals per group to provide robust and reproducible results while adhering to the 3Rs principle (Replacement, Reduction, Refinement) for animal research. Five mice were included in each experimental group. Tumor dimensions, including width (W) and length (L), were measured weekly using calipers, and tumor volumes were calculated using the formula *L* × *W*^2^ × 0.52. Capsanthin was administered intraperitoneally at two doses, which are low (0.036 mg/kg) and high (0.36 mg/kg), twice a week for 4 weeks. At the end of the study, the mice were sacrificed for further analysis.

### Immunohistochemistry

Immunohistochemical analysis was conducted on paraffin-embedded sections of tumors collected from control and Capsanthin-treated mice following sacrifice. Tumor tissues were initially fixed in paraformaldehyde and then processed into thin sections (3 μm). These sections were stained with hematoxylin and eosin to assess morphology, followed by incubation with anti-EZH2 (Cell signaling, #5246) and anti-PD-L1 (GeneTex, #GTX636033) antibodies to evaluate EZH2 and PD-L1 expression levels.

### Statistical analysis

The data represent the mean ± SD and were subjected to analysis by one-way analysis of variance with the Tukey’s tests or Student’s *t*-test for assessing variations among multiple groups. All statistical computations were carried out using GraphPad Prism software 8.0 (La Jolla, CA, USA) on at least three biological replicates for each experiment (check figure legends for details). A *P*-value of <0.05 was considered to reflect a statistically significant difference between values.

## Supplementary information

Raw data

## Data Availability

The data that support the findings of this study are available from the corresponding author upon reasonable request.
