# Supplementary figures and images for: Capsanthin inhibits migration and reduces N-linked glycosylation of PD-L1 via the EZH2-PD-L1 axis in triple-negative breast cancer brain metastasis
Source: Cell Death Discov. 2025 Mar 4;11:85. doi: 10.1038/s41420-025-02368-1 (PMC11880297; doi:10.1038/s41420-025-02368-1)

Fig.3 D

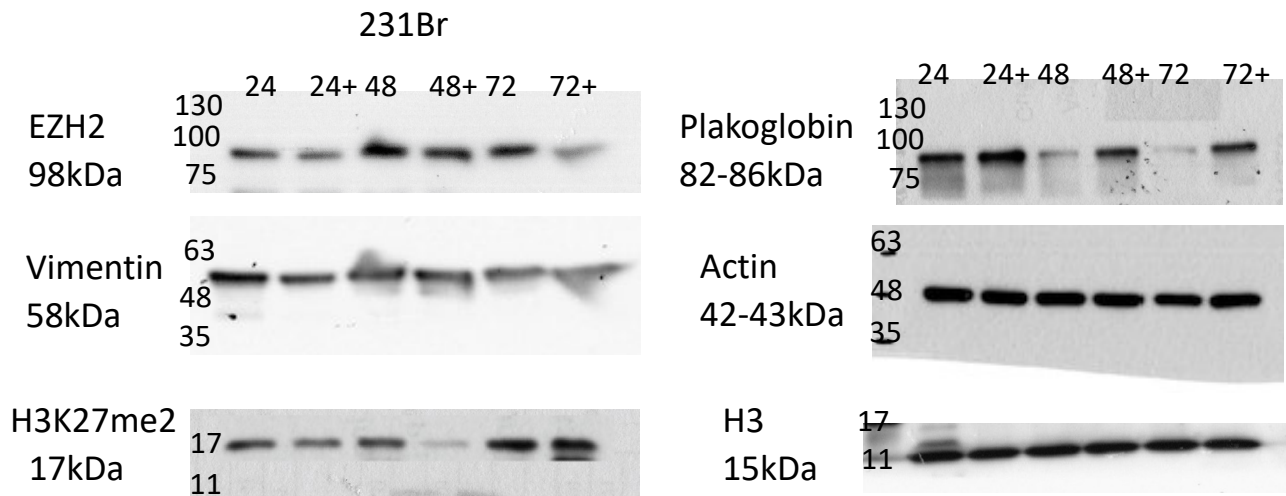

Fig.4C

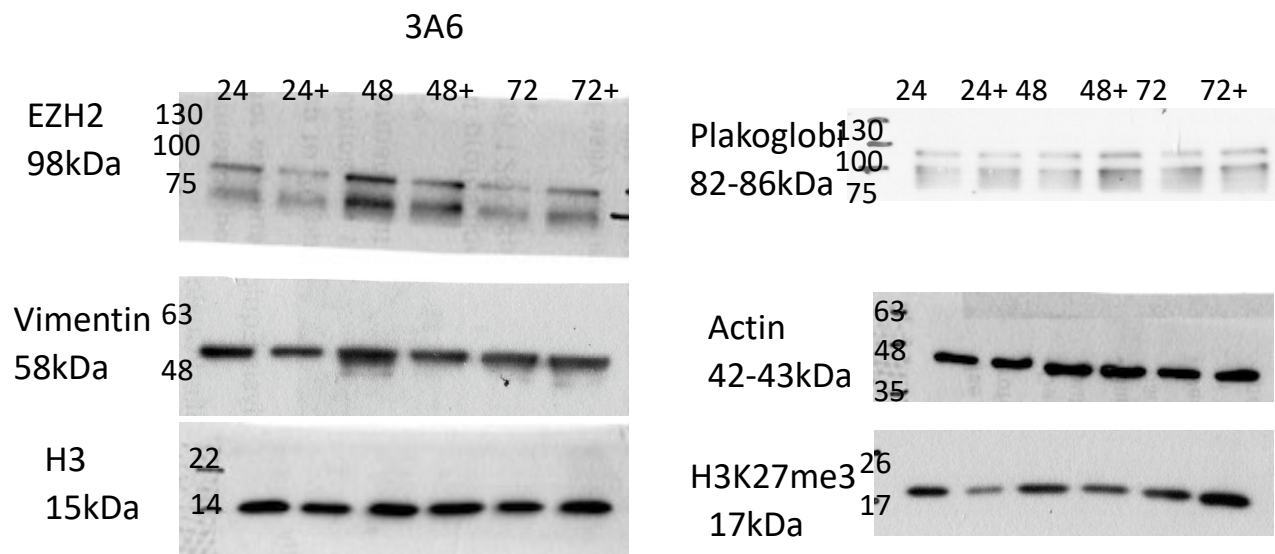

Fig.8A

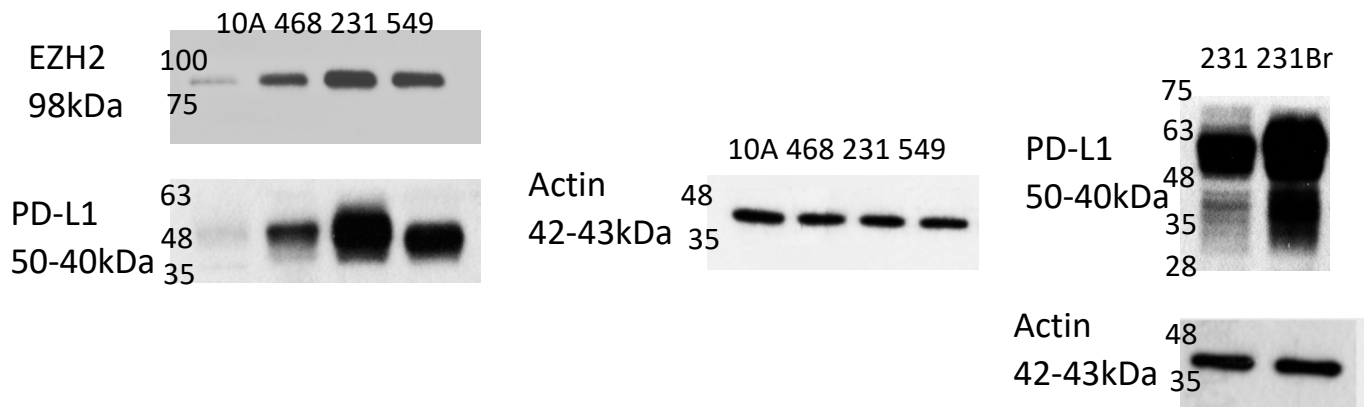

Fig.8B

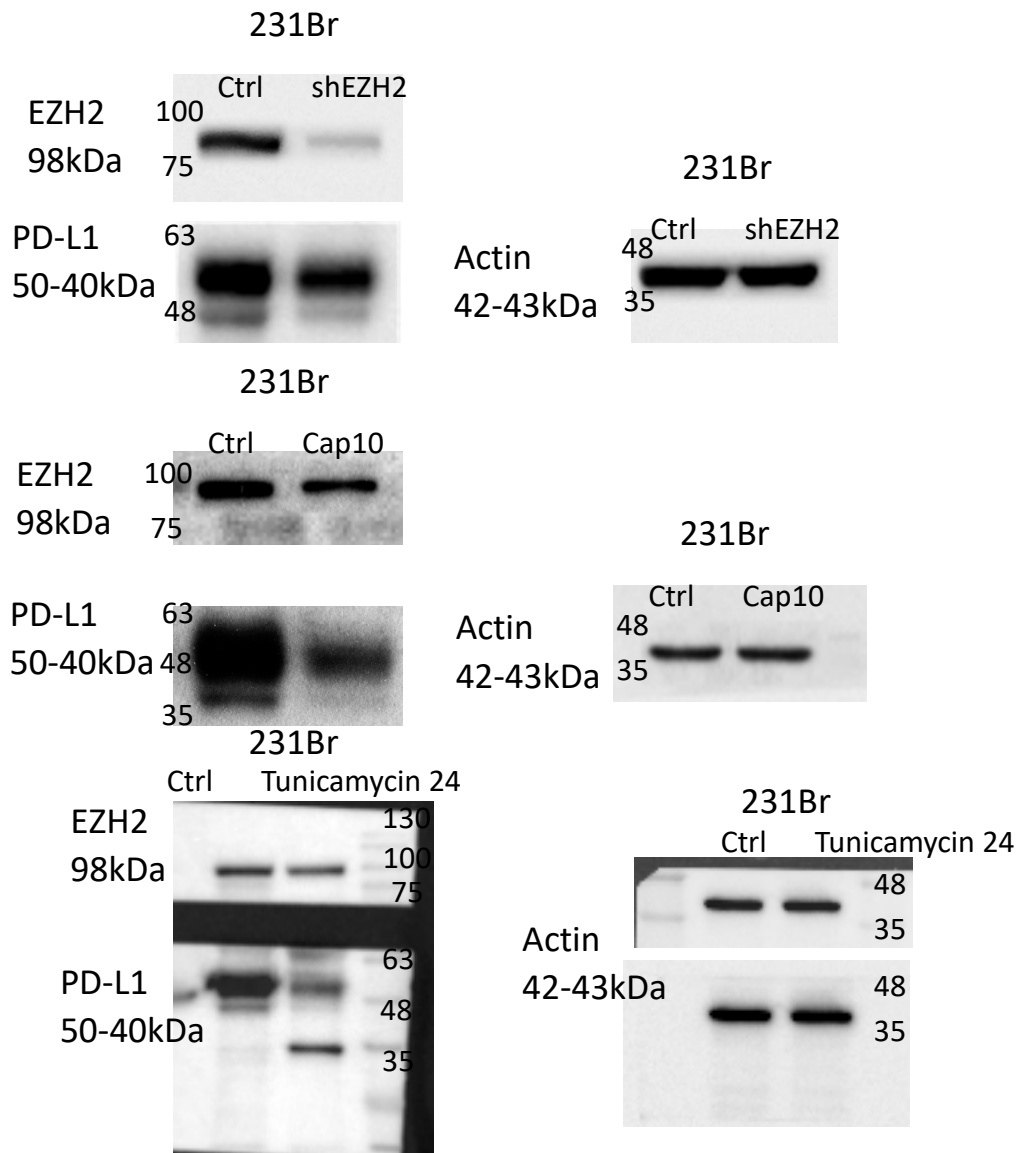

Fig.8C

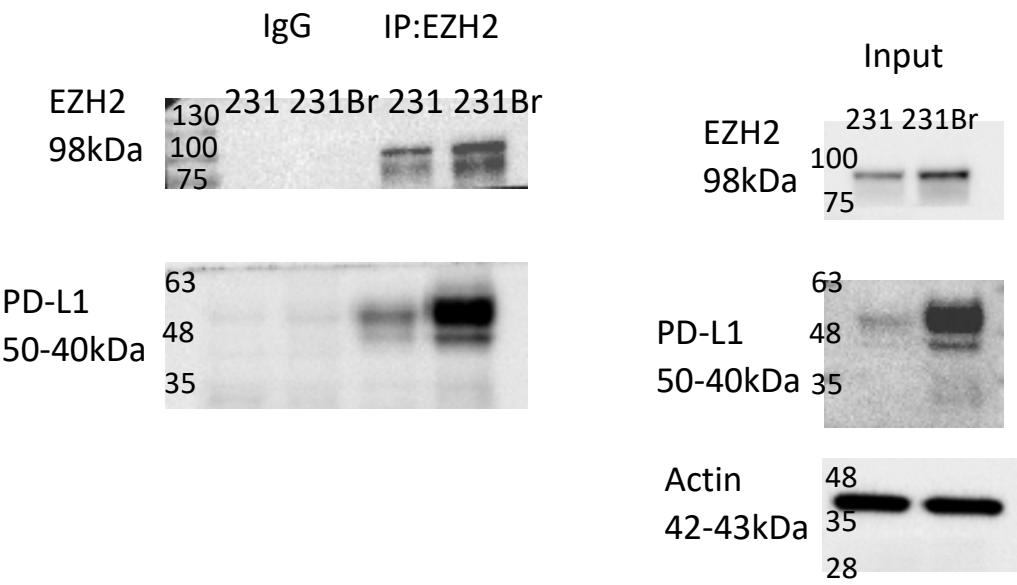

Fig.8D

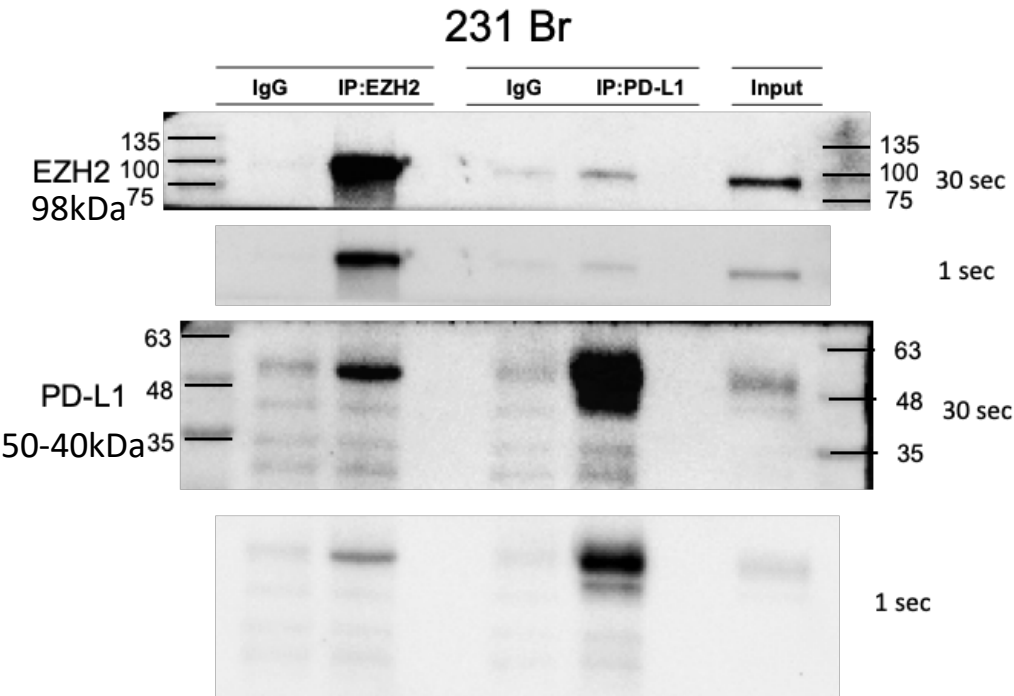

Fig.8E

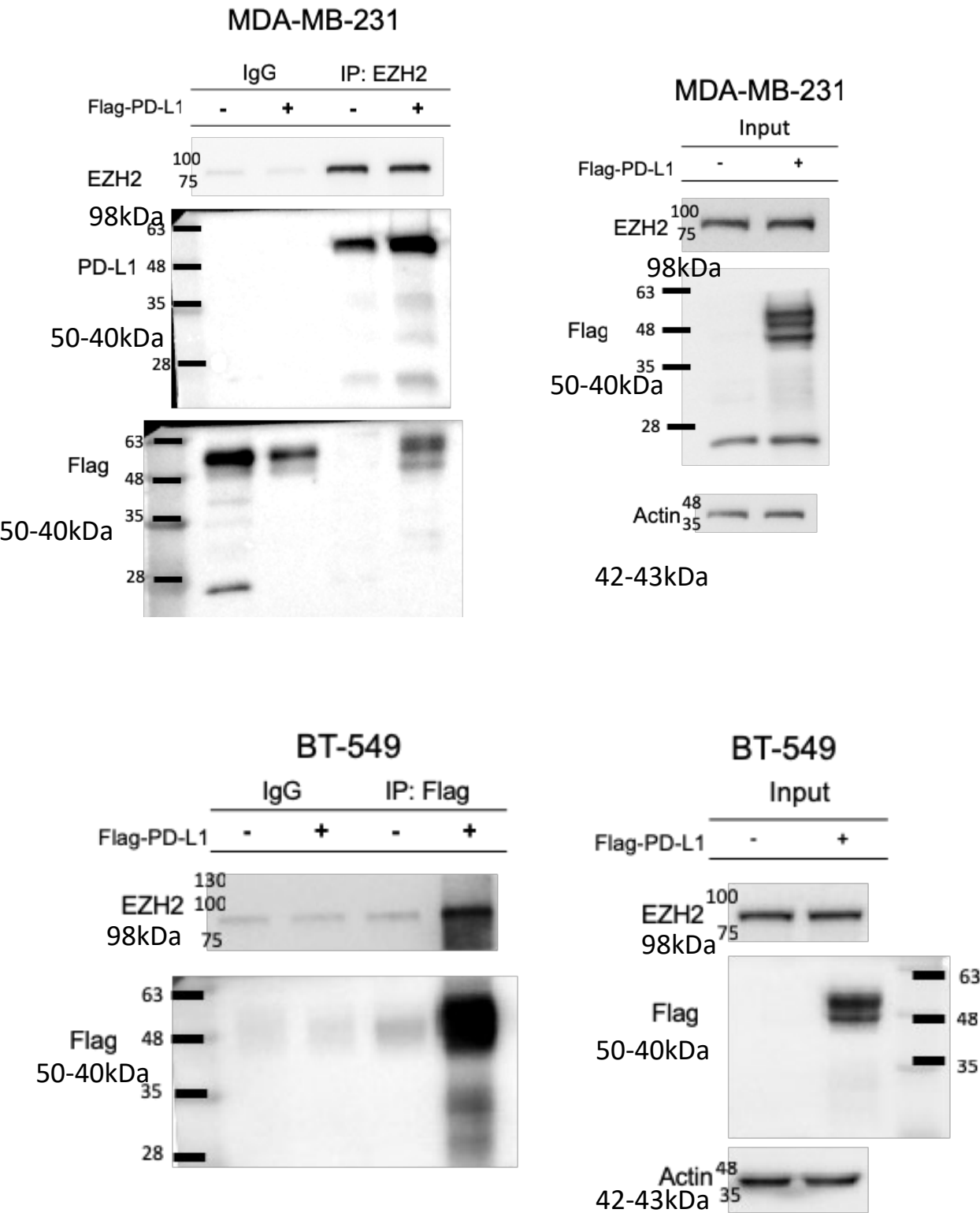

Fig.9E

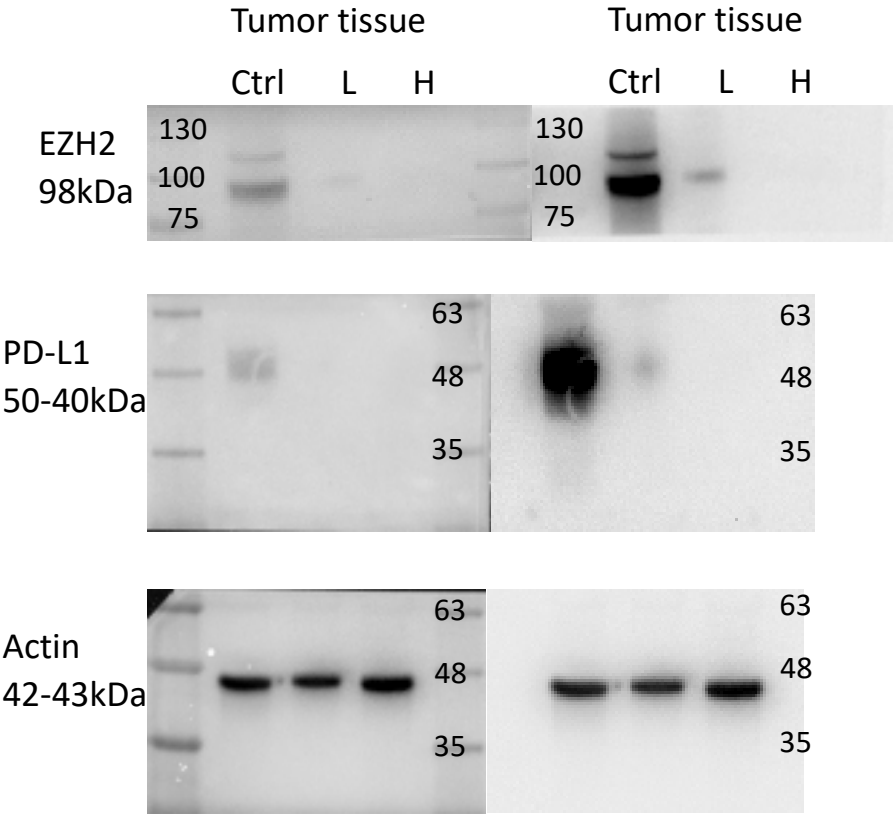

Supplement: Supplementary file 1 — Raw data [file 41420_2025_2368_MOESM1_ESM.pdf]
